# The effects of co-application of organic fertilizer and limestone powder on soil organic carbon and its fractions in rubber plantations: associations with microbial necromass carbon and Fe/Al oxides

**DOI:** 10.3389/fmicb.2026.1970224

**Published:** 2026-10-12

**Authors:** Jifen Yang, Shunjun Geng, Xiaoling Shi, Qianghao Zhang, Chunxia Yang

**Affiliations:** 1 Yunnan Key Laboratory of Sustainable Utilization Research on Rubber Tree, Jinghong, Yunnan, China; 2 Yunnan Institute of Tropical Crops, Jinghong, Yunnan, China

**Keywords:** acidic soils in rubber plantations, Fe/Al oxides, microbial necromass carbon, organic fertilizer and lime, soil organic carbon fractions

## Abstract

**Introduction:**

Rubber plantations serve as significant carbon reservoirs within China’s terrestrial ecosystems. To provide a scientific foundation for improving soil fertility management, this study investigated the response of soil organic carbon and its components in rubber plantations to different soil amendments.

**Methods:**

Soil samples were collected from the 0–20 cm horizon of acidic soils in second-generation rubber plantations in Jinghong City, Yunnan Province. A laboratory incubation experiment was then conducted with four treatments: an unamended control (CK), organic fertilizer alone (T1), limestone powder alone (T2), and a combined organic fertilizer and limestone powder treatment (T3). The effects of these amendments on soil organic carbon and its fractions, iron and aluminum oxides, and microbial necromass carbon were systematically evaluated.

**Results:**

The results demonstrated that under T3, Organic carbon (SOC) increased significantly by 12.80%, relative to CK (*p* < 0.05). Treatments T2 and T3 significantly enhanced microbial necromass carbon (by 6.92–10.90%), fungal necromass carbon (by 6.60–11.32%), and bacterial necromass carbon (by 7.79–9.74%) compared to CK (*p* < 0.05). T3 also significantly increased amorphous (Fe_o_, Al_o_) and complexed (Fe_p_, Al_p_) iron and aluminum oxides *(p* < 0.05). Two-way ANOVA revealed a significant interaction between organic fertilizer and limestone powder on POC, MAOC, MNC, and oxide forms, indicating an interactive regulatory effect on the distribution of carbon fractions and the transformation of mineral forms. Regression and correlation analyses showed microbial necromass carbon and Fe/Al oxides significantly positively correlated with SOC and its fractions, suggesting close associations among necromass accumulation, oxide protection, and SOC fraction changes. VPA showed that the shared explanatory power of microbial and mineral variables for SOC stabilization exceeded their respective pure effects.

**Conclusion:**

In summary, microbial necromass carbon, iron and aluminum oxides, and pH are closely related to soil organic carbon and its components. The combined application of organic fertilizer and limestone powder is highly effective in mitigating soil acidification and increasing SOC, microbial necromass carbon, and iron and aluminum oxides, and can serve as a reference for soil fertility management and carbon pool regulation in acidified soils of tropical rubber plantations.

## 1 Introduction

Soil organic carbon constitutes the largest carbon reservoir in terrestrial ecosystems and is critical to soil fertility, food production, and global climate regulation. Its reserves, composition, and stability directly determine soil productivity and the extent to which its ecological functions are fulfilled (Stockmann et al., 2013). Rubber plantations constitute an important economic forest system in China’s tropical regions while simultaneously serving as a significant carbon sink within the country’s terrestrial ecosystems, thereby playing a vital role in the terrestrial carbon cycle (Zhang et al., 2022). Studies based on long-term flux observations indicate that the net ecosystem exchange (NEE) of rubber plantation ecosystems can reach −911.89 g C m^−2^ a^−1^, demonstrating significant carbon sink capacity (Yang et al., 2023). However, under intensive management practices, soil organic carbon in rubber plantations faces the risk of depletion. The conversion of natural forests has led to reduced litter input, and soil acidification further threatens the stability of carbon pools. This coexistence of “ecosystem carbon sinks” and “soil carbon depletion” reveals a critical shortcoming in current rubber plantation management regarding carbon cycle regulation: the accumulation of biomass carbon has not been effectively converted into stable soil carbon storage. Therefore, effectively promoting organic carbon accumulation and stabilization in rubber plantation management has become a key issue for the sustainable development of tropical agriculture.

The accumulation of soil organic carbon depends not only on the quantity of exogenous organic matter input, but is also mediated by the interplay of multiple mechanisms, including mineral protection, physical encapsulation within soil aggregates, and microbial conversion efficiency (Lorenz and Lal, 2014). In tropical and subtropical acidic soils, iron and aluminum oxides play a particularly significant role in the stabilization of organic carbon. On the one hand, iron oxides form stable organic–mineral complexes with organic carbon through ligand exchange, surface adsorption, and coprecipitation; on the other hand, iron acts as a catalyst to facilitate the conversion of organic carbon into more chemically recalcitrant forms (Tian et al., 2026). Notably, recent research has revealed that soil acidification may promote organic carbon accumulation by enhancing the mineral-protective capacity of iron and aluminum oxides (Yu et al., 2024). This finding provides a new perspective for understanding carbon sequestration pathways in tropical acidic soils, while simultaneously suggesting a complex, nonlinear relationship among acidification, mineral protection, and carbon accumulation. Soil microbial necromass carbon (including bacterial necromass carbon and fungal necromass carbon), as an important component of the stable organic carbon pool, is closely related to the formation and transformation of organic carbon fractions. Indeed, global-scale studies have indicated that microbial necromass carbon can account for more than 51% of the organic carbon in the 0–20 cm soil layer of agricultural soils (Wang et al., 2021), underscoring the dominant role of soil microorganisms in regulating organic carbon dynamics (Angst et al., 2021; Wang et al., 2021). Specifically, microorganisms externally modify plant-derived carbon through the secretion of extracellular enzymes. A portion of the degradation products is incorporated into particulate organic carbon (POC), while small-molecule substrates undergo intracellular turnover to synthesize microbial biomass. Following cell death, microbial necromass bind to soil minerals, thereby forming more stable mineral-associated organic carbon (MAOC) (Angst et al., 2021; Wang et al., 2023). It is worth noting that in tropical rubber plantation soils, microbial necromass carbon accumulation responds significantly to both vegetation type and parent material. Microbial biomass and necromass carbon in the topsoil of rubber plantations are higher than in other vegetation types (Li et al., 2024). However, long-term monoculture and extensive management may alter microbial community structure, thereby potentially affecting the net accumulation of necromass carbon (Wang et al., 2021). Furthermore, widespread acidification of rubber plantation soils (pH < 5.0) may indirectly influence the formation and stabilization of necromass carbon by altering microbial community composition (e.g., the fungal-to-bacterial ratio) and mineral surface properties. Therefore, systematically elucidating the mechanisms of organic carbon accumulation from the dual perspectives of mineral preservation and microbial transformation is of critical importance for the scientific management of carbon pools in agricultural ecosystems.

To address soil acidification and carbon pool degradation in rubber plantations, the judicious application of soil amendments has become a widely adopted management practice. Organic fertilizers and lime are among the most commonly used amendments for soil improvement. Organic fertilizers are rich in easily decomposable organic matter and a variety of nutrients. When applied, they replenish the soil with active carbon sources and may promote the accumulation of carbon from microbial necromass by stimulating the metabolic activity of soil microorganisms (Cheng et al., 2025). Long-term field trials in Hainan Province have shown that replacing chemical fertilizers with organic fertilizers significantly increases SOC and pH levels. It also enhances SOC stability, as indicated by changes in carbon mineralization potential and enzyme activity (Xu et al., 2026). As a traditional soil amendment, limestone powder acts through mechanisms distinct from those of acidification. On the one hand, it raises soil pH, thereby alleviating acid stress on microorganisms and promoting the accumulation of microbial biomass and necromass carbon. On the other hand, the increase in pH elevates the content of amorphous and complexed Fe/Al oxides. These active minerals carry abundant positive surface charges and can tightly bind negatively charged organic carbon through ligand exchange and surface complexation, forming stable organo-mineral complexes and thereby enhancing the stability of newly introduced organic carbon (Fang et al., 2019; Shen et al., 2024). Acidification and liming influence SOC through different stages of the carbon cycle: acidification primarily affects the degree of mineral protection of existing carbon stocks, whereas liming promotes the accumulation of newly added carbon by improving the microbial environment and activating mineral surfaces (Abdelaziz et al., 2026). Together, these two processes determine the net balance of soil organic carbon in rubber plantations. In long-term field experiments on subtropical acidic soils, the combined application of organic fertilizer and lime significantly increased organic carbon accumulation in microaggregates, and the stability of carbon in macroaggregates under organic fertilizer treatment was primarily associated with amorphous iron oxides (Trivedi et al., 2021). Therefore, analyzing the patterns of soil organic carbon changes under the application of soil conditioners and identifying the associated factors holds significant theoretical value for the targeted enhancement of soil carbon stocks. Although some studies have begun to explore the response of organic carbon in rubber plantations to soil conditioners, (Liu et al., 2023). However, little is known about how soil amendments influence organic carbon accumulation and stabilization by regulating microbial necromass carbon and Fe/Al oxides, and comprehensive studies on this specific ecosystem—rubber plantations—remain insufficient. In particular, in-depth comparisons are lacking regarding the differences in organic carbon fraction distribution under different amendment treatments in tropical rubber plantation soils, as well as the patterns of their association with microbial necromass carbon and Fe/Al oxides—specifically, how mineral retention and microbial necromass carbon accumulation respond to different types of amendments.

To address this knowledge gap, this study focused on a typical acidified rubber plantation in Xishuangbanna, Yunnan Province, China. Through a 60-day indoor cultivation experiment, the study aimed to investigate the response characteristics of soil organic carbon and its components in the acidified rubber plantation under the influence of different soil conditioners, and to identify the correlation patterns between microbial necromass carbon and iron-aluminum oxides. Therefore, by analyzing the statistical associations between these variables and organic carbon fractions, this study provides exploratory evidence for understanding the relative roles of microbial transformation and mineral stabilization in organic carbon accumulation, rather than directly verifying the synergistic mechanisms between the two. Specifically, this study addresses the following three questions: (1) How do soil organic carbon and its fractions respond to the application of different soil conditioners? (2) What are the response patterns of microbial necromass carbon and iron-aluminum oxides to different soil amendments? What statistical associations exist between these two components and organic carbon fractions? (3) Based on the aforementioned association patterns, can key factors closely related to changes in organic carbon fractions be identified? What testable hypotheses do these association patterns provide for understanding the stabilization pathways of soil organic carbon in rubber plantations? Ultimately, this study aims to provide insights into the potential pathways through which soil conditioners influence soil organic carbon in tropical rubber plantations by examining two measurable dimensions of association: microbial necromass carbon accumulation and the protective effects of iron and aluminum oxides.

## 2 Materials and methods

### 2.1 Study site overview

The test soil was an acidic red soil collected from the second-generation rubber plantation at the Fourth Brigade of Jiangbei, Jinghong City, Yunnan Province, China (22°2′23.424″N, 100°47′45.343″E). The study site has a tropical monsoon climate, with a mean annual temperature of 23 °C and a mean annual precipitation of 1,400 mm. Prior to analysis, soil samples were air-dried, manually freed of roots, stones, and other debris, and sieved through a 5-mm mesh. The basic physicochemical properties of the experimental soil were as follows: pH 5.20, organic carbon 19.93 g·kg^−1^, total nitrogen 1.82 g·kg^−1^, available phosphorus 2.43 mg·kg^−1^, alkali-hydrolyzable nitrogen 124.65 mg·kg^−1^, and available potassium 26.37 mg·kg^−1^.

### 2.2 Experimental design

The experiment comprised four treatments: a control with no fertilizer (CK), organic fertilizer alone (T1), limestone powder alone (T2), and a combined application of organic fertilizer and limestone powder (T3). The organic fertilizer used in this study was supplied by Xishuangbanna Kangming Biotechnology Co., Ltd. and produced by fermenting sheep manure. It had an organic matter content of ≥30%, a total carbon content of 18.40%, carbon input was 1.14 g C kg^−1^, a pH of 7.56, and a total nutrient content (N + P₂O₅ + K₂O) of ≥4%, The test modifier was limestone powder (main component: calcium carbonate, CaCO₃ ≥ 98.00%, purity ≥ 98.00%, particle size ≥ 600. mesh, purchased from Ning’er Jiabao Putty Powder Factory).

Three replicates were established for each treatment, and soil samples were collected 60 days after cultivation. Organic fertilizer was applied at a rate of 6.20 g·kg^−1^ dry soil (Zhang et al., 2025), Lime was applied at a rate of 1.80 g·kg^−1^ dry soil (Hu et al., 2016).

Acidic soil samples (300.00 g) were thoroughly mixed with each treatment and transferred to culture bottles. Soil moisture content was adjusted to 70% of field capacity using deionized water. The bottles were sealed with plastic film, with a small perforation left at the center to allow gas exchange while minimizing moisture loss. The culture bottles were then placed in a constant-temperature incubator at 25 °C. Samples were weighed every 3 days, and deionized water was supplemented to maintain consistent moisture content throughout the incubation period. Destructive sampling was performed after 60 days of incubation.

### 2.3 Determination of soil parameters

Soil pH, total nitrogen (TN), soil organic carbon (SOC), available phosphorus (AP), alkali-hydrolyzable nitrogen (AN), and available potassium (AK) were quantified using methods described in previous studies (Serafim et al., 2023). Soil pH was measured with a pH meter in a 1:2.5 (w/v) soil–water suspension. Soil organic carbon content was determined by the potassium dichromate external heating method, the sample was pretreated with 1 mol·L^−1^ HCl until no more bubbles were produced, then washed with deionized water until the pH reached neutral before measurement to eliminate potential interference from inorganic carbon. Total nitrogen (TN) was determined by the Kjeldahl method, and alkali-hydrolyzable nitrogen (AN) was assessed by the alkaline hydrolysis diffusion method. Available phosphorus (AP) was measured with an AA3 continuous-flow analyzer after extraction with NH₄F solution. Available potassium (AK) was extracted with 1 mol·L^−1^ neutral ammonium acetate (NH₄OAc) solution and measured using a flame photometer.

Particulate organic carbon (POC) and mineral-associated organic carbon (MAOC) were determined following the method described in previous studies (Cambardella and Elliott, 1992). Air-dried soil (20 g, <2 mm) was weighed into a centrifuge tube, and 60 mL of sodium hexametaphosphate solution (5 g·L^−1^) was added. The mixture was shaken at 180 r·min^−1^ for 18 h at 25 °C. After dispersion, the suspension was passed through a 53 μm sieve, and the material retained on the sieve was rinsed repeatedly with deionized water until the effluent was clear. The fraction retained on the sieve (>53 μm) was defined as particulate organic carbon (POC), while the fraction passing through the sieve (<53 μm) was defined as mineral-associated organic carbon (MAOC). Both fractions were oven-dried at 60 °C, ground to pass through a 100-mesh sieve, and subsequently analyzed for organic carbon content. The POC and MAOC contents were calculated using the following equation:

$$\begin{array}{rr} \mathrm{POC}\;(g\;{\mathrm{kg}}^{-1}) & =\mathrm{POM}-C\;\text{content}\;(g\;{\mathrm{kg}}^{-1})\times \frac{\mathrm{POM}\;\text{mass}\;(g)}{\text{Soil mass}\;(g)}\\ \text{MAOC}\;(g\;{\mathrm{kg}}^{-1}) & =\text{MAOM}-C\;\text{content}\;(g\;{\mathrm{kg}}^{-1})\times \frac{\text{MAOM mass}\;(g)}{\text{Soil mass}\;(g)} \end{array}$$

Amino sugar quantification in soil was performed following the protocol described in reference (Zhang and Amelung, 1996). After hydrolysis and purification, the four amino sugar derivatives—glucosamine (GluN), mannosamine (ManN), galactosamine (GalN), and muramic acid (MurA)—were determined using high-performance liquid chromatography with fluorescence detection (HPLC-FLD). Microbial necromass carbon was calculated according to the method of Liang et al. (2017) using the following equation:

$$\begin{array}{rr} \mathrm{BNC} & =\text{MurA}\times 45\\ \mathrm{FNC} & =(\text{GluN}/179.17-2\times \text{MurA}/251.23)\times 179.17\times 9\\ \mathrm{MNC} & =\mathrm{FNC}+\mathrm{BNC}\\ \mathrm{AS} & =\text{GluN}+\text{ManN}+\text{GalN}+\text{MurA} \end{array}$$

Where FNC and BNC represent fungal and bacterial necromass carbon, respectively; 179.17 and 251.23 are the molecular weights of glucosamine (GluN) and muramic acid (MurA), respectively; and 45 and 9 are the conversion factors from MurA to bacterial necromass carbon and from GluN to fungal necromass carbon, respectively.

Soil iron and aluminum oxides were quantified following the protocol described in reference (Mckeague and Day, 1966).

#### 2.3.1 Free iron and aluminum oxides (Fe_d_, Al_d_)—DCB extraction method

Extraction was performed using a sodium dithionite–sodium citrate–sodium bicarbonate (DCB) solution. Air-dried soil (0.50 g) passed through a 0.25 mm sieve was placed in a 50 mL centrifuge tube. Then, 10 mL of 0.3 mol·L^−1^ sodium citrate solution and 1.5 mL of 1 mol·L^−1^ sodium bicarbonate solution were added. The mixture was heated in a water bath at 80 °C until the temperature stabilized, after which 0.25 g of sodium dithionite (Na₂S₂O₄) was added. The suspension was stirred at constant temperature for 15 min, cooled, and centrifuged. The supernatant was transferred to a volumetric flask. The extraction was repeated 1–2 times, and the supernatants were combined, diluted to volume, and prepared for analysis.

#### 2.3.2 Amorphous iron and aluminum oxides (Fe_o_, Al_o_)—Acidic ammonium oxalate extraction method

Extraction was performed using 0.2 mol·L^−1^ acidic ammonium oxalate buffer (Tamm’s reagent, pH 3.0–3.2). Air-dried soil (0.50 g) passed through a 0.25 mm sieve was weighed, and 50 mL of ammonium oxalate buffer was added at a soil-to-solution ratio of 1:50. After capping, the vessel was placed in a double-layered cloth bag lined with infrared-black fabric to protect it from light and shaken at 200 rpm on a constant-temperature shaker at 25 °C for 2 h. After extraction, the mixture was centrifuged, and the supernatant was filtered through a 0.45 μm membrane before analysis.

#### 2.3.3 Complexed iron and aluminum oxides (Fe_p_, Al_p_)—Sodium pyrophosphate extraction method

Extraction was performed using 0.1 mol·L^−1^ sodium pyrophosphate solution (pH 8.50–10.00). Air-dried soil (2.50 g) passed through a 0.25 mm sieve was weighed, and 50 mL of sodium pyrophosphate solution was added at a soil-to-solution ratio of 1:20. The mixture was shaken for 2 h on a constant-temperature shaker at 25 °C and then allowed to stand for 14–16 h. After centrifugation, the supernatant was filtered through a 0.45 μm membrane and analyzed.

#### 2.3.4 ICP-OES analysis conditions

The concentrations of Fe and Al in the extract are determined using inductively coupled plasma optical emission spectrometry (ICP-OES). Recommended detection wavelengths: Fe 259.94 nm, Al 396.15 nm. Instrument operating conditions: RF power 1,300–1,400 W, nebulizer flow rate 0.70–0.80 L·min^−1^, cooling gas flow rate 12–15 L·min^−1^, auxiliary gas flow rate 0.2–1.0 L·min^−1^.

### 2.4 Data analysis

All experimental data were preprocessed using Excel 2020. SPSS 21.0 software was used to perform a two-way analysis of variance (ANOVA) to examine the main effects and interactions of organic fertilizer and lime on soil organic carbon and its components, microbial necromass carbon, and the forms of iron and aluminum oxides. Subsequently, a one-way ANOVA was conducted to compare the various indicators among different treatment groups, and Duncan’s multiple range test was used for post-hoc comparisons at a significance level of *p* < 0.05. Prior to conducting the analysis of variance, all datasets were subjected to the Shapiro–Wilk normality test and Levene’s test for homogeneity of variances. All data met the assumptions of normality and homogeneity of variances (*p* > 0.05).

Linear regression analysis was used to examine the relationships between microbial necromass carbon and organic carbon, as well as between soil organic carbon and iron and aluminum oxides (Fe_o_, Al_o_, Fe_p_, Al_p_).

Redundancy analysis (RDA) was used to explore the multivariate relationships between pH, iron and aluminum oxides, and microbial residual carbon variables and SOC and its components. Prior to analysis, the variance inflation factor (VIF) was calculated for all explanatory variables, and variables with a VIF > 10 were excluded to control for multicollinearity. A forward selection procedure was used to screen for a subset of variables with significant explanatory power; significance was assessed using a Monte Carlo replacement test (999 replacements). Adjusted *R*^2^ rather than raw *R*^2^ is reported to account for the influence of the number of predictor variables relative to the sample size. The significance of the global model was assessed via a replacement test; if the global model did not reach the significance level, the RDA results were used only for exploratory reference and not as a basis for statistical inference.

Variance decomposition analysis (VPA) was used to quantify the relative contributions of the microbial variable groups (MNC, FNC, BNC) and mineral variable groups (Fe_o_, Al_o_, Fe_p_, Al_p_) to the variation in SOC components. The shared fractions from the VPA reflect information overlap between the two groups of variables rather than biological interactions; therefore, they are provided for exploratory reference only.

Graphical representations were generated using Origin 2021. Redundancy analysis (RDA) was performed using Canoco 5, and variance partitioning analysis was conducted using the vegan package in R.

## 3 Results

### 3.1 Main effects and interactions of organic fertilizer and lime

The results of the two-way analysis of variance indicated that organic fertilizer and limestone powder had differing effects on soil organic carbon and its components, microbial necromass carbon, and the forms of iron and aluminum oxides (Table 1). Organic fertilizer had a significant effect on, Fe_d_, and Al_d_ (*p* < 0.05), with the effects on MAOC, Al_o_, and Al_p_ reaching the highly significant level (*p* < 0.01 or *p* < 0.001); limestone powder had a significant effect on Al_p_ (*p* < 0.05), with the effects on SOC, MAOC, MNC, Fe_p_, Fe_d_ and Al_d_ reaching the highly significant level (*p* < 0.01 or *p* < 0.001).

**Table 1:** Two-factor analysis of variance for soil organic carbon and its components, and iron and aluminum oxides under the influence of organic fertilizer and lime.

Factors	Organic fertilizer	*p*	Lime	*p*	Organic fertilizer × lime	*p*
SOC	2.50 NS	0.15	17.63^**^	0.003	0.31 NS	0.59
POC	0.79 NS	0.40	5.27 NS	0.051	12.63^**^	0.007
MAOC	13.22^**^	0.007	14.46^**^	0.005	27.28^***^	<0.001
MNC	0.13 NS	0.73	33.53^***^	<0.001	6.80^*^	0.031
Fe_o_	4.89 NS	0.058	0.57 NS	0.47	4.97 NS	0.056
Al_o_	31.39^***^	<0.001	1.19 NS	0.31	29.33^***^	<0.001
Fe_p_	1.00 NS	0.35	26.92^***^	<0.001	3.74 NS	0.09
Al_p_	15.99^**^	0.004	6.00^*^	0.04	25.52^***^	<0.001
Fe_d_	10.38^*^	0.012	29.72^***^	<0.001	5.87^*^	0.042
Al_d_	5.48^*^	0.047	26.22^***^	<0.001	3.26 NS	0.11

^*^, ^**^, and ^***^ indicate significant effects at the *p* < 0.05, *p* < 0.01, and *p* < 0.001 levels, respectively; NS indicates no significant effect.

Notably, the interaction between organic fertilizer and limestone powder was significant for POC (*p* < 0.01), MAOC (*p* < 0.001), MNC (*p* < 0.01), Al_o_ (*p* < 0.001), Al_p_ (*p* < 0.001), and Fe_d_ (*p* < 0.05), indicating that their combined effect on these parameters is not a simple additive relationship.

### 3.2 Soil organic carbon and its fractions

Soil organic carbon (SOC) content was significantly higher under T3 than under CK, with an increase of 12.80% (*p* < 0.05) (Table 2). Regarding organic carbon fractions, all three amendment treatments increased particulate organic carbon (POC) content compared with the control (CK), with significant increases observed under T1 and T2 (*p* < 0.05). Mineral-associated organic carbon (MAOC) content was significantly higher under T2 than under CK, with an increase of 10.00% (*p* < 0.05, Figure 1).

**Table 2:** Physicochemical properties of soil under different amendments.

Treatment	pH	SOC	TN	C/N	Al^3+^
CK	4.46 ± 0.05d	17.12 ± 0.58c	1.74 ± 0.03c	9.84 ± 0.47bc	9.72 ± 0.31a
T1	4.69 ± 0.01c	17.93 ± 0.98bc	1.84 ± 0.01a	9.73 ± 0.51c	6.18 ± 0.23b
T2	4.90 ± 0.08b	18.92 ± 0.39ab	1.78 ± 0.01b	10.65 ± 0.15a	2.72 ± 0.13c
T3	5.23 ± 0.04a	19.31 ± 0.53a	1.84 ± 0.01a	10.51 ± 0.28ab	1.82 ± 0.10d

Fe_o_, amorphous iron; MNC, microbial necromass carbon; FNC, fungal necromass carbon; Al_p_, aluminum in complex form; Fe_p_, iron in complex form; BNC, bacterial necromass carbon; Al_o_, amorphous aluminum.

**Figure 1:**
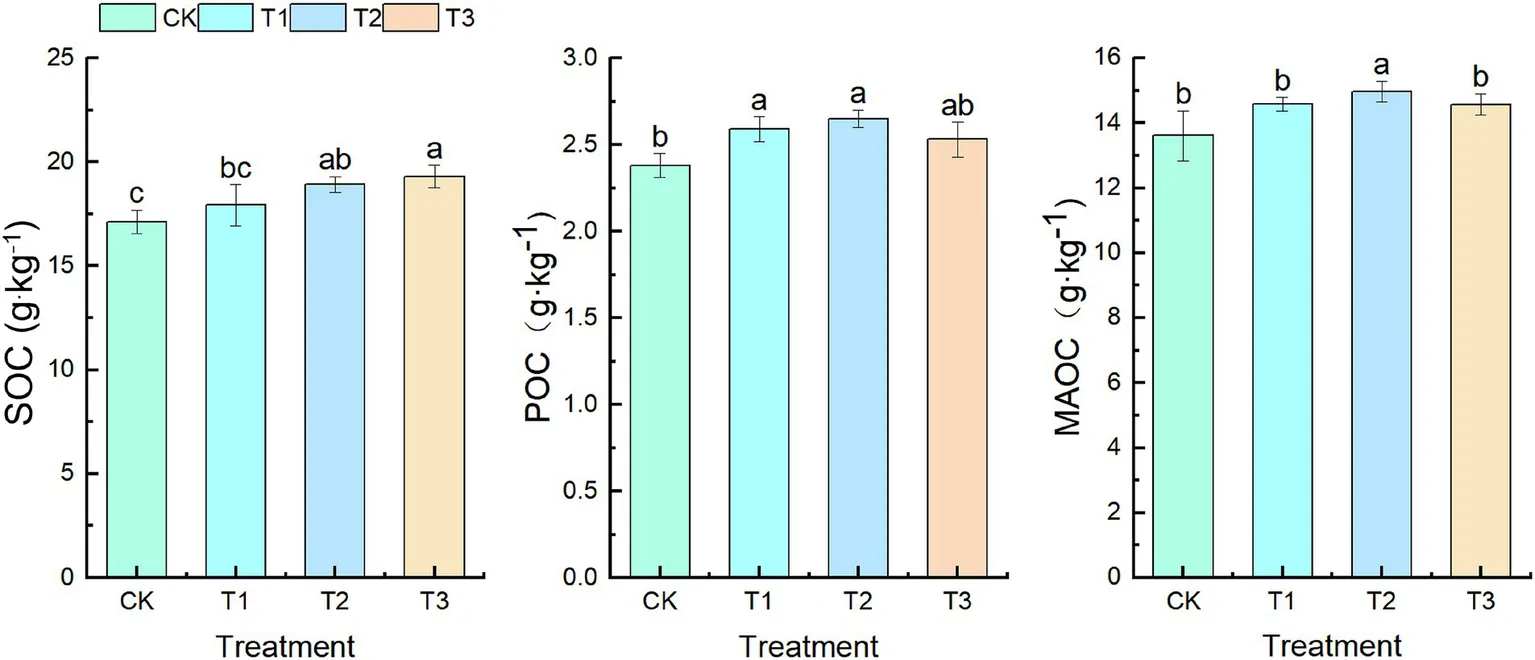
Soil organic carbon and its components under different amendments.

### 3.3 Soil microbial necromass carbon and their contribution to SOC

The application of different amendments altered the contents of amino sugars and microbial necromass carbon. Compared with the control (CK), both T2 and T3 treatments significantly increased total amino sugar content, with increases of 12.35 and 6.20%, respectively (*p* < 0.05). Fungal necromass carbon (FNC) content varied significantly among treatments (*p* < 0.05), with T2 and T3 showing increases of 11.32 and 6.60%, respectively, relative to CK. Bacterial necromass carbon (BNC) content was significantly increased by all three treatments (T1, T2, and T3), with increases of 5.20%, 9.74%, and 7.80%, respectively (*p* < 0.05). Total microbial necromass carbon (MNC) content also differed significantly among treatments (*p* < 0.05), with T2 and T3 showing significant increases of 10.90 and 6.92%, respectively, compared with CK (Figure 2).

**Figure 2:**
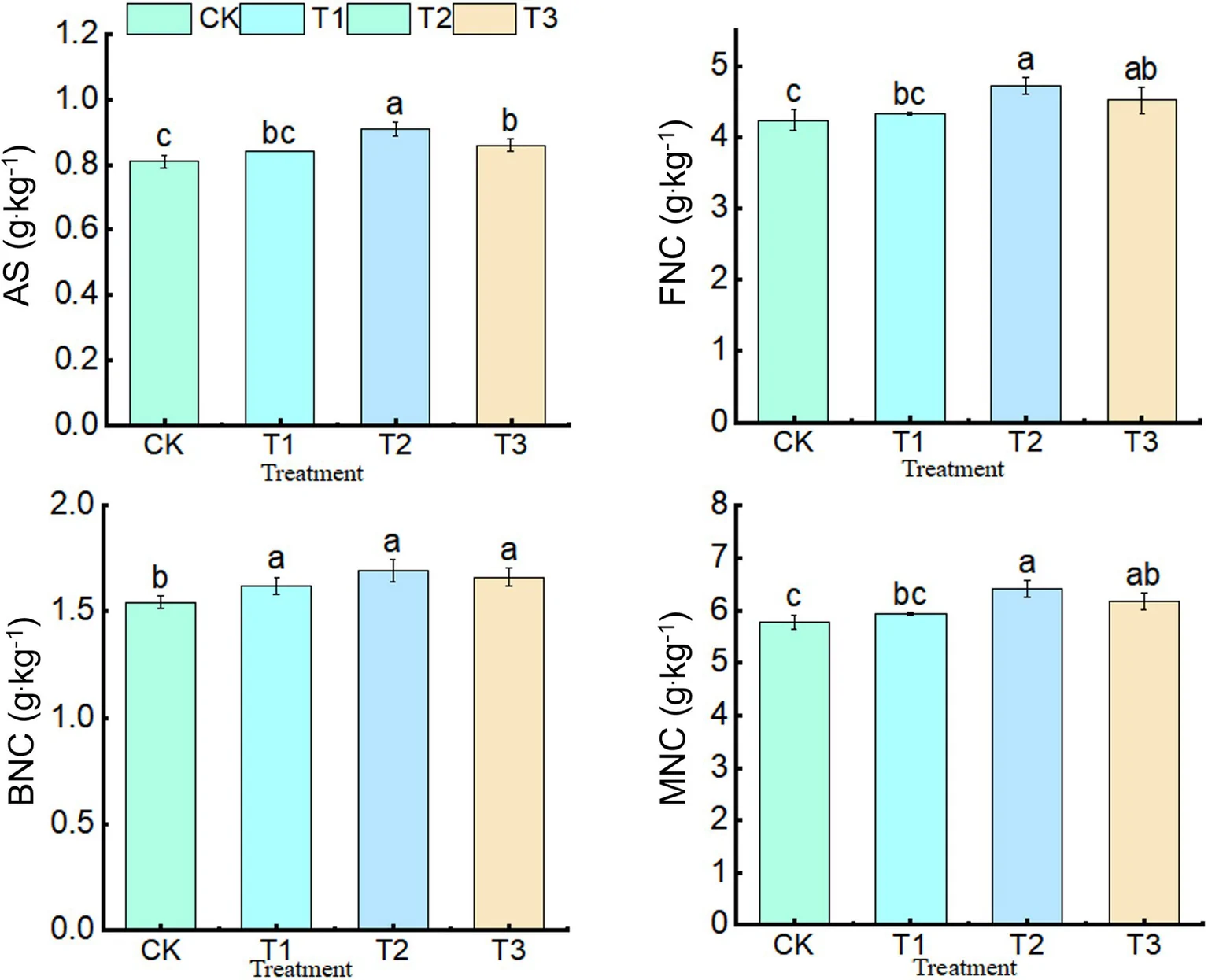
Carbon content in soil microbial necromass under different amendments. AS, amino sugars; MNC, microbial necromass carbon; FNC, fungal necromass carbon; BNC, bacterial necromass carbon.

No significant differences were observed among treatments in the ratios of microbial necromass carbon to soil organic carbon (MNC/SOC), fungal necromass carbon to SOC (FNC/SOC), or bacterial necromass carbon to SOC (BNC/SOC) (*p* > 0.05, Figure 3). Across all treatments, the proportion of fungal necromass carbon (FNC) relative to organic carbon was significantly higher than that of bacterial necromass carbon (BNC) relative to organic carbon.

**Figure 3:**
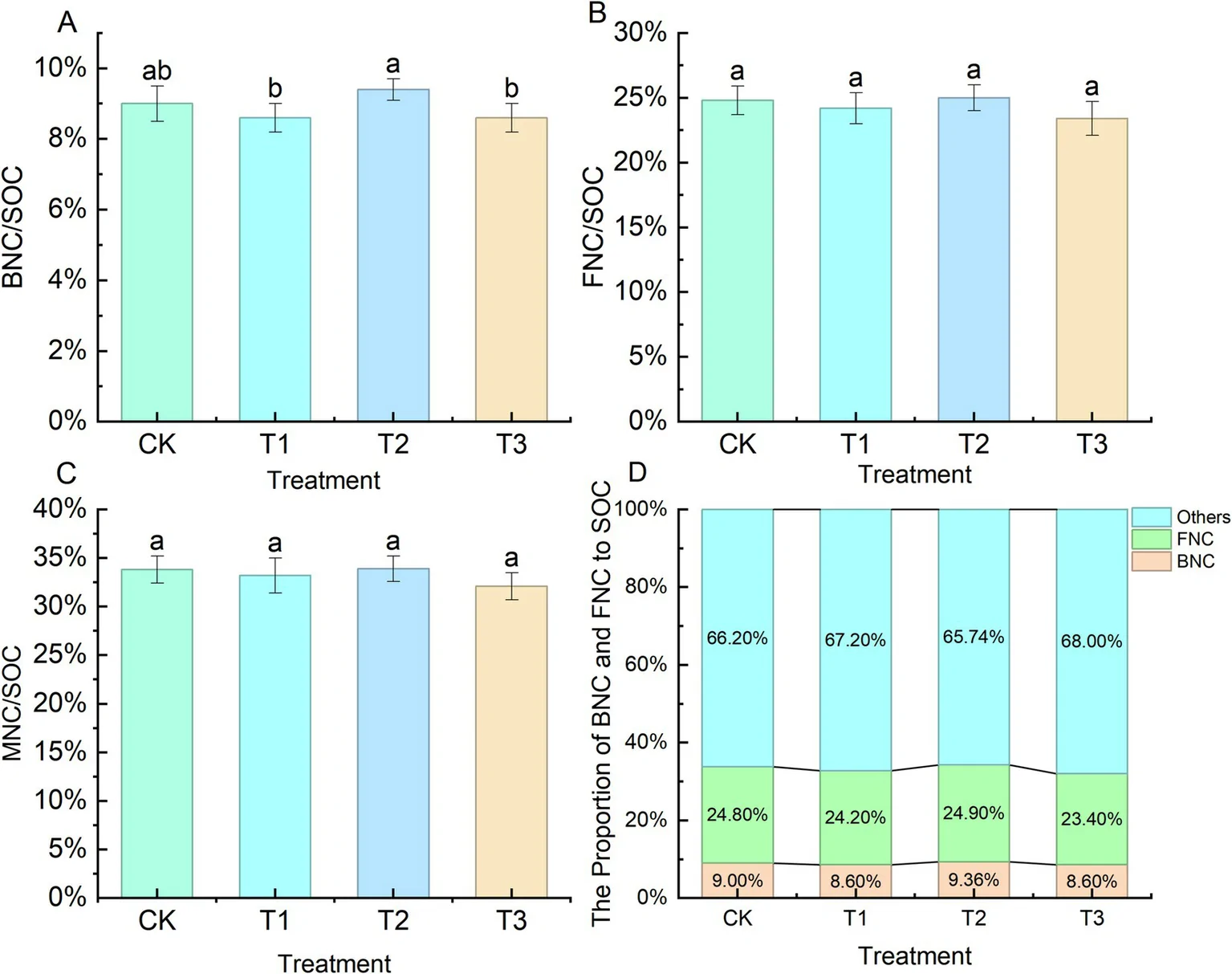
Proportion of different carbon sources to total soil organic carbon. **(A)** Proportion of BNC to SOC. **(B)** Proportion of FNC to SOC. **(C)** Proportion of MNC to SOC. **(D)** Proportion of BNC and FNC to SOC.

### 3.4 Iron and aluminum oxides in soils

The application of different soil amendments altered the contents of various iron and aluminum oxide fractions. Compared with the control (CK), the T1 treatment significantly increased free iron (Fe_d_) and free aluminum (Al_d_) contents by 12.53 and 12.89%, respectively (*p* < 0.05). Complexed aluminum (Al_p_) content was significantly increased by all three treatments, with increases of 9.32% (T1), 7.74% (T2), and 6.64% (T3) relative to CK (*p* < 0.05), whereas complexed iron (Fe_p_) was significantly increased only by T3 (7.74%; *p* < 0.05). Amorphous iron (Fe_o_) and amorphous aluminum (Al_o_) contents varied significantly among treatments (*p* < 0.05). Compared with the control (CK), treatment T3 significantly increased amorphous iron (Fe_o_) content by 7.41% (*p* < 0.05), while Al_o_ was significantly increased by all three treatments, with increases of 10.38% (T1), 9.43% (T2), and 16.04% (T3) relative to CK (*p* < 0.05, Figure 4).

**Figure 4:**
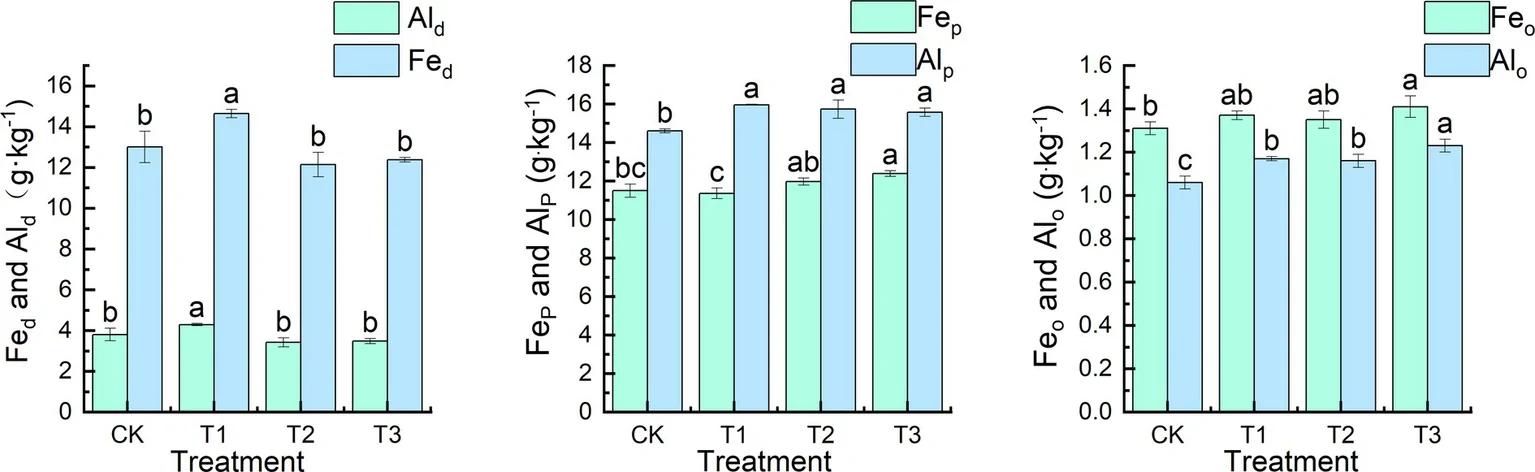
Iron and aluminum oxide content in soil under different amendments. Fe_d_, free iron; Al_d_, free aluminum; Fe_p_, complexed iron; Al_p_, complexed aluminum; Fe_o_, amorphous iron; Al_o_, amorphous aluminum.

### 3.5 Correlation analysis of soil organic carbon and its components with microbial necromass carbon and iron-aluminum oxides

Regression analysis revealed that soil organic carbon was significantly positively correlated with complexed iron (Fe_p_), amorphous iron (Fe_o_), and amorphous aluminum (Al_o_) (*p* < 0.05), but negatively correlated with free iron (Fe_d_) and free aluminum (Al_d_) (Figure 5). Fungal necromass carbon (FNC), bacterial necromass carbon (BNC), and total microbial necromass carbon (MNC) all exhibited significant positive correlations with soil organic carbon (*p* < 0.05, Figure 6).

**Figure 5:**
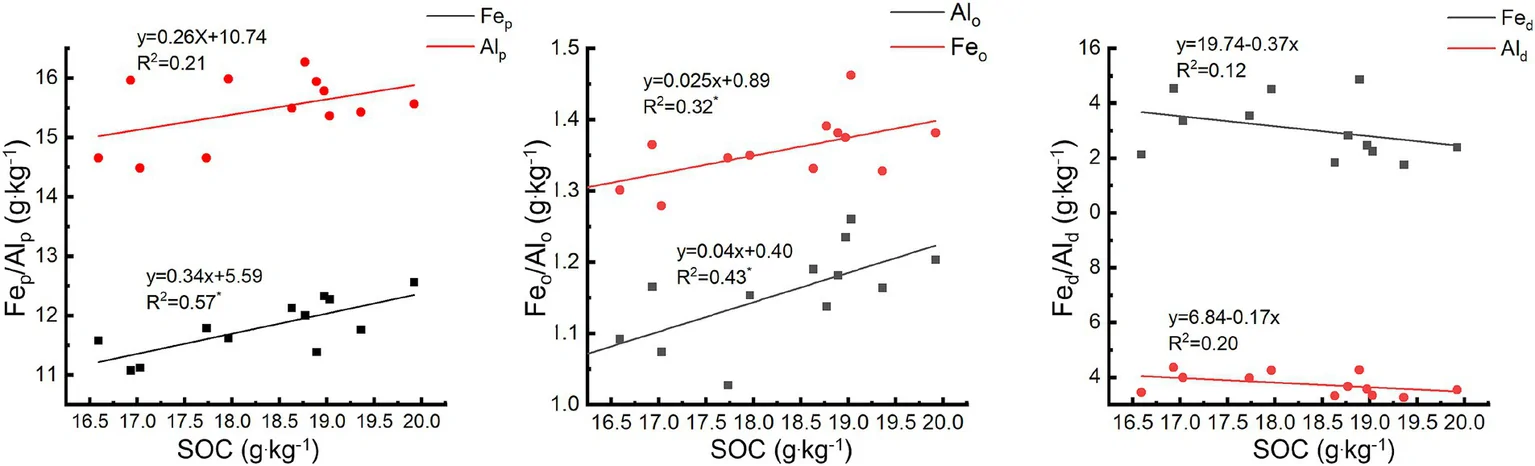
Correlation between iron-aluminum oxides and soil organic carbon.

**Figure 6:**
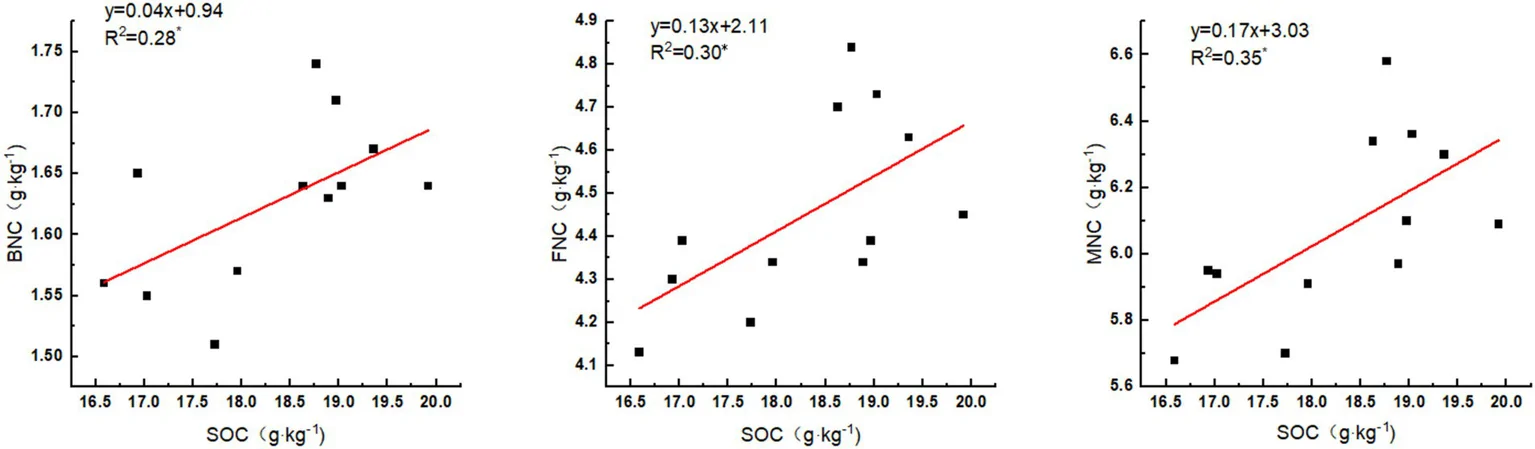
Correlation between soil microbial necromass carbon and organic carbon.

The results of the correlation analysis indicate that SOC is significantly correlated with MNC, BNC, FNC, and Al_o_ (*p* < 0.05) and extremely significantly correlated with pH and Fe_p_ (*p* < 0.01); POC is significantly correlated with MNC, BNC, Al_o_, and Al_p_ (*p* < 0.05); and MAOC is significantly correlated with MNC, BNC, FNC, Fe_o_, and Al_p_ (*p* < 0.05, Figure 7).

**Figure 7:**
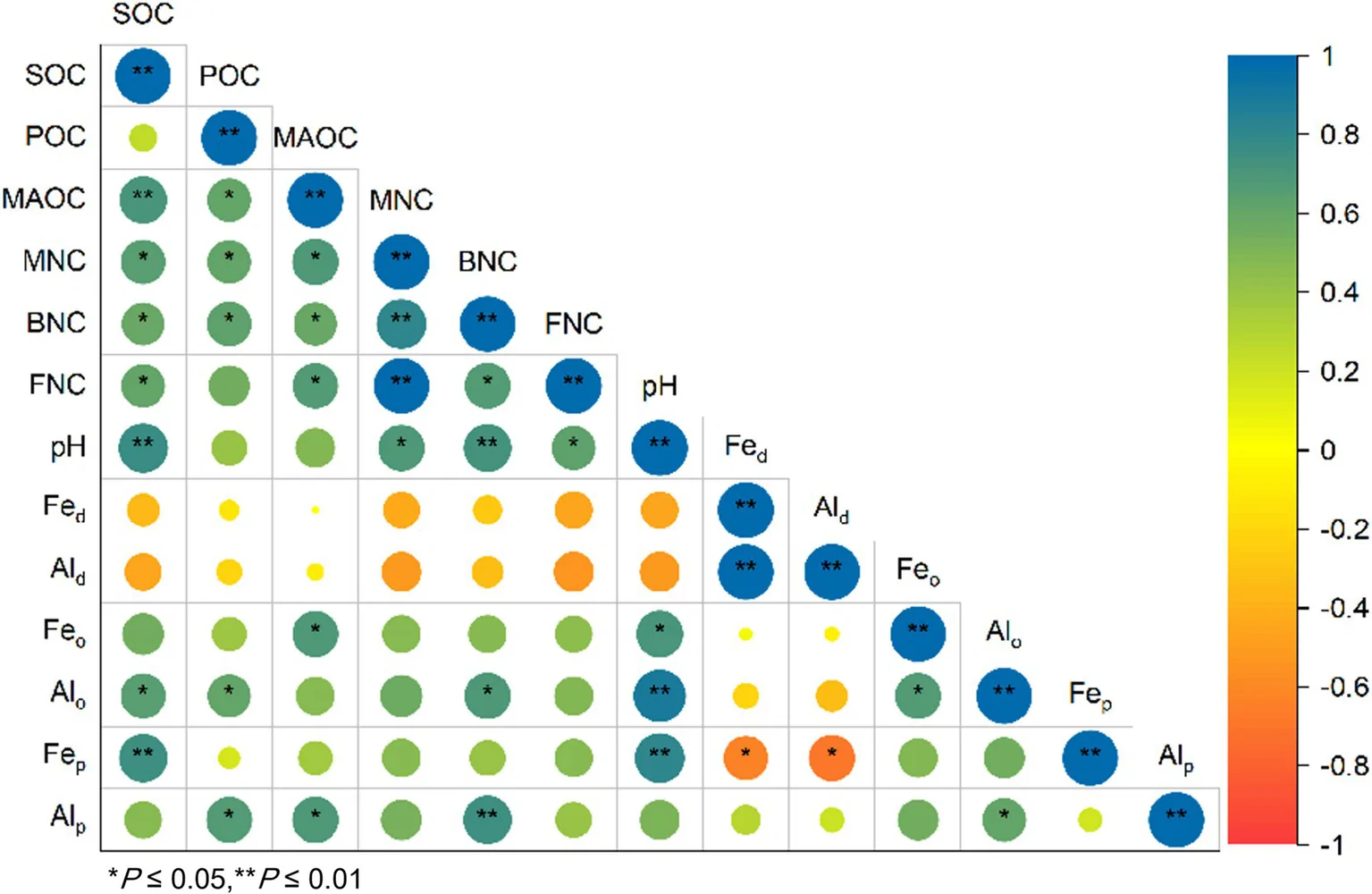
Correlations among soil organic carbon fractions, microbial necromass carbon, iron and aluminum oxides, and pH. POC, particulate organic carbon; MAOC, mineral-associated organic carbon; MNC, microbial necromass carbon; BNC, bacterial necromass carbon; FNC, fungal necromass carbon; Fe_o_/Al_o_, amorphous Fe/Al; Fe_p_/Al_p_, complexed Fe/Al; Fe_d_/Al_d_, free Fe/Al. Circle color and size represent Pearson correlation coefficients; blue indicates positive correlation and red indicates negative correlation. ^*^p < 0.05, ^**^p < 0.01.

RDA analysis showed that the first two axes collectively explained 75.23% of the variance (RDA1: 66.53%, RDA2: 8.70%), with an adjusted *R*^2^ of 0.299. However, the global model permutation test did not reach statistical significance (*p* = 0.281, 999 permutations), indicating that the set of measured explanatory variables as a whole did not significantly explain the variation in SOC components. Therefore, the RDA ordination plot is presented for exploratory purposes only, to visualize the relative relationships among variables, and should not be used as a basis for statistical inference (Figure 8).

**Figure 8:**
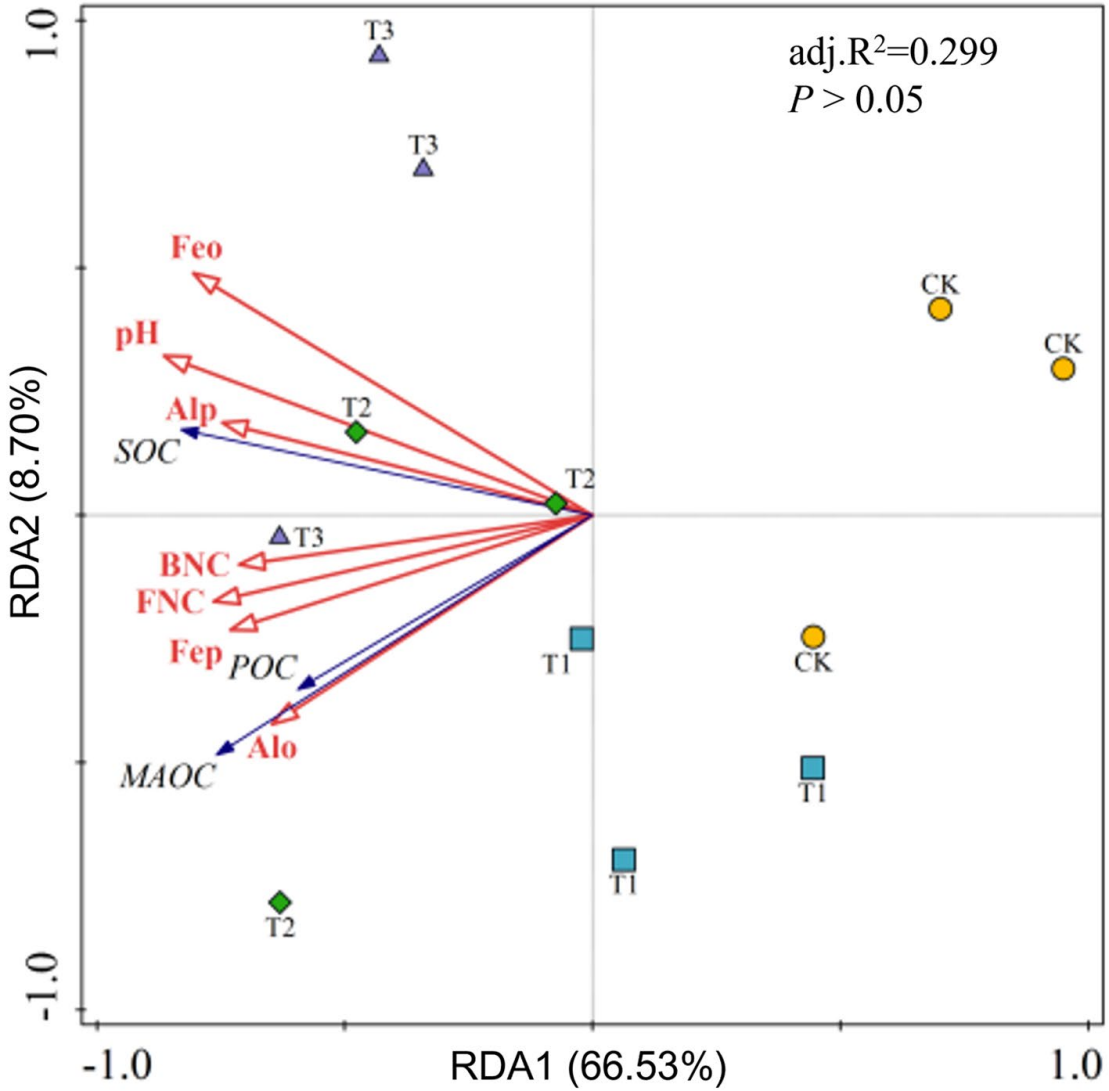
Redundancy analysis of soil properties, microbial necromass carbon, iron and aluminum oxides, and organic carbon and its fractions. Fe_o_, amorphous iron; FNC, fungal necromass carbon; Al_p_, aluminum in complex form; Fe_p_, iron in complex form; BNC, bacterial necromass carbon; Al_o_, amorphous aluminum.

Variance decomposition analysis (VPA) illustrates the relative contributions of microbial and mineral variables to the variability of soil organic carbon fractions (Figure 9). For total soil organic carbon (SOC), the independent contribution of mineral variables was 8.11%, while the shared contribution of microbial and mineral variables was 39.46%. For particulate organic carbon (POC), the shared contribution of microbial and mineral variables was 48.40%. For mineral-associated organic carbon (MAOC), the independent contribution rates of microbial and mineral variables were 9.00 and 10.06%, respectively, with a shared contribution of 26.84%. These results indicate that microbial and mineral variables share a significant portion of the variance in organic carbon components, reflecting information overlap between the two sets of variables.

**Figure 9:**
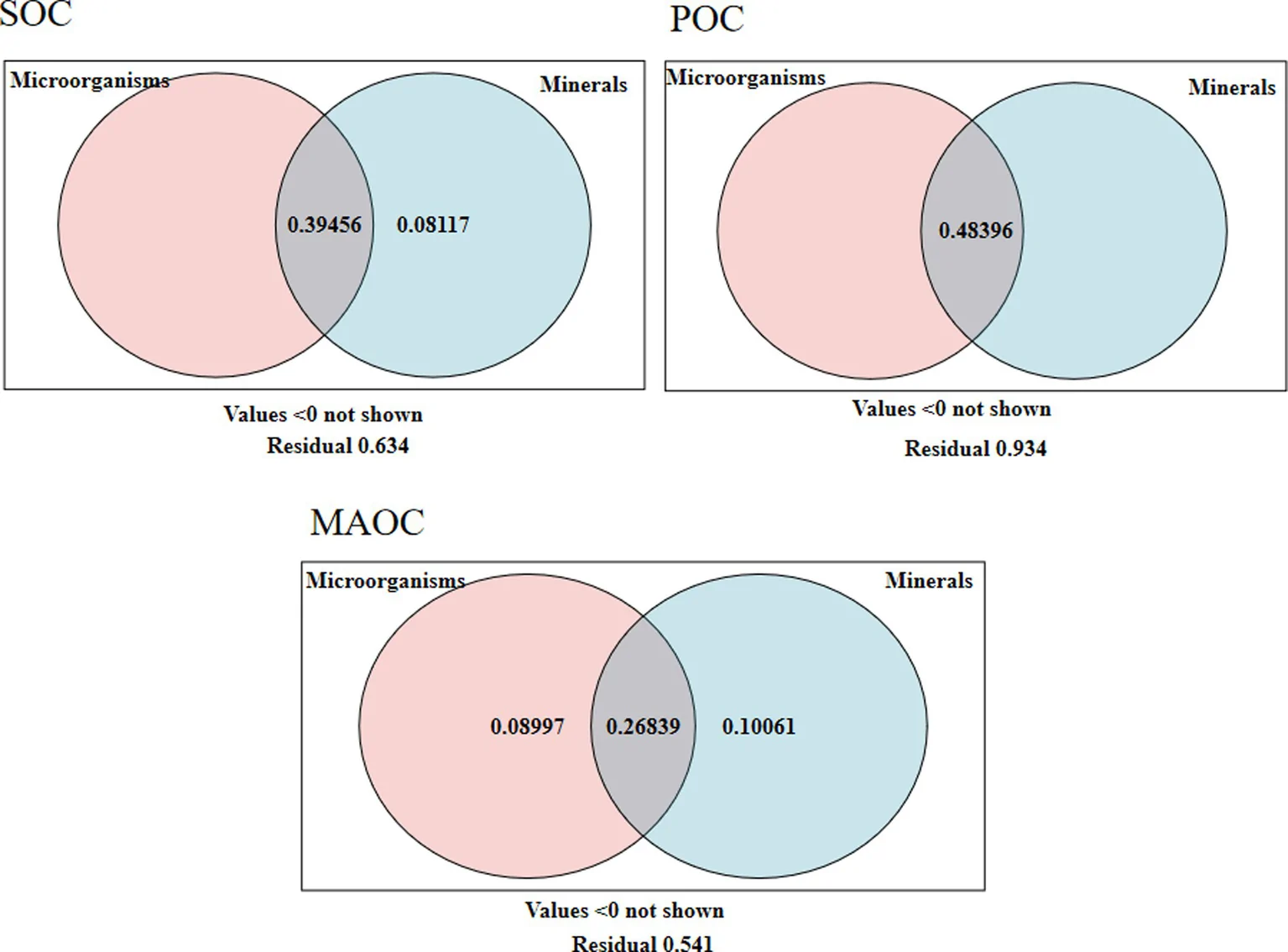
Analysis of variance for microorganisms, minerals, and soil organic carbon and its fractions.

## 4 Discussion

### 4.1 Responses of soil organic carbon and its fractions to different amendments

The application of soil conditioners exerted significant effects on soil organic carbon and its fractions. In this study, both T2 (limestone powder) and T3 (organic fertilizer + limestone powder) treatments significantly increased soil organic carbon content, with T3 exhibiting the most pronounced increase. Lime application alleviated aluminum toxicity by elevating soil pH, which in turn increased exchangeable calcium content in the soil (Schroeder et al., 2024). Notably, among all treatments, T3 exhibited the greatest efficacy in raising soil pH and reducing exchangeable Al^3+^ content. As an alkaline soil amendment, lime acts primarily by releasing base cations, including calcium (Ca^2+^) and magnesium (Mg^2+^), which displace exchangeable H^+^ and Al^3+^ from soil colloid surfaces. This displacement process effectively reduces the active concentrations of these acidic species and neutralizes exchangeable acidity (Muhammad et al., 2015). Organic fertilizers possess the dual properties of alkaline substances and humus-rich colloidal matter with strong buffering capacity, effectively enhancing the acid–base buffering capacity of soils (Riwandi et al., 2017). Furthermore, the humus derived from organic fertilizer decomposition contains abundant functional groups, such as carboxyl and phenolic hydroxyl groups, which can form stable complexes with aluminum ions, thereby reducing free Al^3+^ activity (Cai et al., 2021). When applied in combination, lime rapidly raises soil pH and precipitates aluminum ions, while the humus in organic fertilizer sustains the elevated pH over the long term. This synergistic effect alleviates aluminum toxicity constraints on microbial activity, enhances microbial metabolism and activity, and consequently promotes soil organic carbon accumulation. Consistent with this, previous studies have reported that soil organic carbon content under combined organic fertilizer and lime application is significantly higher than under single-treatment conditions (Trivedi et al., 2021), findings that align well with the results of the present study.

With respect to organic carbon fractions, the T1 and T2 treatments significantly increased POC content. POC consists primarily of partially degraded plant necromass and microbial metabolites and serves as an important source of carbon and energy for soil microorganisms. In this study, the increase in POC under T1 treatment likely resulted mainly from the direct carbon input from organic fertilizer, which provided an exogenous carbon source for the POC pool (Mao et al., 2024). The increase in POC under T2 treatment may be related to the ability of lime to improve soil structure and enhance aggregate formation. Previous field studies have shown that lime application can promote root biomass input by improving soil structure, thereby increasing the active organic carbon pool (Carmeis Filho et al., 2017). However, it should be noted that the present study was a laboratory incubation experiment without planted vegetation; therefore, the increase in POC under T2 treatment did not result from root biomass input, but may be related to lime-induced improvements in soil structure and the activation of mineral surfaces. The above explanation regarding root biomass input is based solely on literature inference and requires further validation through field trials. Notably, T2 treatment also significantly increased mineral-associated organic carbon (MAOC) content. This may be attributed to the role of calcium ions (Ca^2+^) as a key chemical bridge in MAOC formation. Previous studies have demonstrated that lime application increases soil Ca^2+^ content, which is closely associated with the accumulation of calcium-bonded carbon. Through chemical cementation, Ca^2+^ promotes the binding of organic carbon to mineral surfaces, thereby enhancing soil organic carbon stability (Briedis et al., 2012). However, although T3 increased total organic carbon, its effect on MAOC was less pronounced than that of T2. This suggests that the use of a single amendment, namely lime alone, may have a more direct and targeted effect on optimizing the MAOC formation pathway.

Microbial necromass carbon is an important component of the stable organic carbon pool and plays a critical role in the long-term sequestration of soil organic carbon (Liang et al., 2017). In this study, the T2 and T3 treatments significantly increased microbial necromass carbon content, whereas the T1 treatment did not reach statistical significance. These results indicate that lime application is closely associated with the accumulation of soil microbial necromass carbon. On the one hand, lime effectively raised the pH of the acidic rubber plantation soil, alleviated acid stress, and improved the microbial habitat, thereby potentially increasing microbial biomass and its turnover rate (Briedis et al., 2012; Yang et al., 2026). On the other hand, lime may have provided physicochemical protection for microbial necromass by promoting soil aggregate formation and enhancing Ca^2+^-mediated organo-mineral interactions, thereby retarding their further decomposition and facilitating the net accumulation of necromass carbon in soil (Hu et al., 2024). Notably, the application of organic fertilizer alone (T1) did not significantly increase microbial necromass carbon (MNC). This may be attributed to two factors. First, in acidic soils without pH amendment, microbial activity remains constrained by low pH and Al^3+^ toxicity, resulting in low microbial carbon use efficiency (CUE) and consequently low necromass conversion efficiency (Schroeder et al., 2024). Second, the readily decomposable organic matter supplied by organic fertilizer may be preferentially channeled into respiratory metabolism rather than being converted into stable microbial necromass (Chen et al., 2020). Studies have shown that when exogenous organic matter is introduced, microbial carbon use efficiency (CUE) may decrease, causing more carbon to be directed toward respiration rather than growth (Zhang et al., 2024). The ratio at which microorganisms allocate absorbed carbon between growth and respiration determines the final amount of litter formed; this process is also regulated by multiple factors, including substrate quality and nutrient availability (Mei et al., 2025). Furthermore, while fungi are relatively dominant in acidic environments and their necromass exhibit high chemical stability, the reduction in necromass formation resulting from an overall decrease in carbon use efficiency (CUE), coupled with potential re-decomposition processes, means that simply increasing the input of exogenous organic matter without improving soil pH conditions often fails to effectively promote the net accumulation of microbial necromass carbon (Moran-Rodas et al., 2023).

### 4.2 Regulation of soil iron and aluminum oxides by different amendments

Iron and aluminum oxides play a pivotal role in the accumulation of organic carbon in acidic soils through mineral protection mechanisms (Yihui et al.). In this study, the application of different amendments exerted differential effects on the speciation of iron (Fe) and aluminum (Al) in rubber plantation soils. The T1 treatment significantly increased the content of free iron and aluminum (Fe_d_ and Al_d_). Free iron and aluminum oxides are generally considered the more stable mineral phases; their increased abundance may provide additional reactive sites for the subsequent formation of complexed and amorphous Fe/Al forms, thereby enhancing the soil’s capacity for organic carbon sequestration (Huang et al., 2026). Following organic fertilizer application, microbial decomposition produces organic acids and phenolic compounds that can promote the activation and transformation of iron and aluminum oxides in soil through various mechanisms (Xu, 1998). Studies have demonstrated that low-molecular-weight organic acids can activate iron oxides through acid-solubilization, complexation-solubilization, and reduction-activation mechanisms (Nworie et al., 2017).

In the present study, both lime-containing treatments (T2 and T3) significantly increased complexed iron (Fe_p_) content relative to the control (CK), while the T3 treatment also significantly elevated the contents of amorphous iron (Fe_o_) and amorphous aluminum (Al_o_). Results indicate that the treatment with organic fertilizer plus limestone powder has the strongest regulatory effect on the mineral forms of iron and aluminum in soil. Studies have shown that amorphous and complexed iron and aluminum oxides exhibit high reactivity in soil and possess a large specific surface area; they can form stable complexes with organic matter through adsorption and coprecipitation, and represent the primary forms of mineral-bound organic carbon in acidic soils (Briedis et al., 2012).

In this study, the T3 treatment creates a microenvironment conducive to the close interaction among “minerals, organic matter, and microorganisms” by simultaneously providing an organic carbon source (organic fertilizer) and a regulator (limestone powder), thereby promoting the accumulation of active iron and aluminum mineral forms. The increased amorphous iron and aluminum content raised the number of active sites on soil mineral surfaces, providing more binding sites for organic carbon sequestration. Concurrently, the elevated content of complexed iron and aluminum indicated the formation of more stable Fe/Al–organic complexes, which further inhibited the biodegradation of organic carbon (Boudot et al., 1989). The synergistic effect of these two mechanisms suggests that the T3 treatment may be superior to single-amendment applications in terms of soil carbon sequestration capacity.

### 4.3 Correlation analysis of soil organic carbon and its components with microbial remnant carbon and iron-aluminum oxides

In this study, pH was found to be significantly positively correlated with soil organic carbon and its fractions. In acidified rubber plantation soils, acidification may promote the dissolution of iron and aluminum oxides, leading to the desorption and release of organic carbon (Shen et al., 2024). The application of lime and organic fertilizers raised soil pH, thereby enhancing the binding of iron and aluminum oxides to organic carbon (Wen et al., 2014). Notably, the positive effect of pH observed in this study may be closely related to the phase transformation of Fe/Al oxides—as pH shifts from acidic toward neutral, the contents of amorphous (Fe_o_, Al_o_) and complexed (Fe_p_, Al_p_) iron and aluminum typically increase, and these active mineral phases serve as the primary carriers for organic carbon adsorption.

Collectively, these results indicate that iron and aluminum oxides govern the storage, turnover, and mineralization of organic carbon in these soils (Rasmussen et al., 2006). Iron promotes soil organic carbon accumulation through three primary mechanisms: promoting soil aggregate formation, adsorbing and coprecipitating with dissolved organic carbon, and modulating microbial activity (Huang et al., 2023; Liu et al., 2024). Similarly, aluminum can adsorb to active mineral surfaces or coprecipitate with the soil solid phase, thereby enhancing the stability of SOC (Porras, 2017). Together, Fe–Al oxides accelerate the accumulation of organically bound iron (OC–Fe) and play a critical role in soil organic carbon sequestration (Wang et al., 2024). Bacterial and fungal necromass are important constituents of the stable soil organic carbon pool (Schweigert et al., 2015). In this study, microbial necromass carbon (MNC, FNC, and BNC) was significantly positively correlated with SOC fractions, with fungal necromass carbon (FNC) exhibiting a stronger correlation than bacterial necromass carbon (BNC). Fungal cell walls are composed primarily of recalcitrant polymers such as chitin, and their mineralization and turnover rates in soil are significantly lower than those of other microbial groups (Kögel-Knabner, 2002). Moreover, fungal hyphal networks can physically bind to soil aggregates, providing a physical protective barrier that promotes the conversion of fungal necromass into stable carbon pools (Daynes et al., 2012). In this experiment, treatments with higher organic matter inputs (T1 and T3) significantly promoted fungal biomass growth; the resulting dead necromass were subsequently incorporated into soil aggregates and the mineral-associated organic carbon pool, further enhancing soil organic carbon accumulation.

### 4.4 Outlook

Through *in vitro* cultivation experiments, this study preliminarily revealed the effects of different soil conditioners on the organic carbon composition and the transformation of Fe/Al oxides in rubber plantation soils, and tentatively suggested a possible association between mineral- and microbial-derived carbon pools in organic carbon allocation. These findings provide preliminary guidance for carbon pool management in acidic rubber plantation soils. However, this study has the following limitations, which need to be addressed in future research: (1) Limitations of the carbon balance. This study did not monitor CO₂-C releases during the incubation period. Therefore, the observed increase in SOC reflects a net accumulation effect (including exogenous carbon inputs, incomplete decomposition of added organic matter, and changes in pre-existing soil carbon) rather than the stabilization efficiency of newly introduced carbon. (2) Limitations regarding sample size. The sample size in this study was small; the results of multivariate analysis should be considered exploratory and hypothesis-generating and require further validation in future studies with larger sample sizes. (3) Limitations regarding microbial characteristics. This study did not directly measure microbial community structure and function; microbial necromass carbon served only as a cumulative indicator of the microbial carbon pool, rather than a direct indicator of microbial community characteristics. Given these limitations, future studies should combine long-term field experiments with laboratory cultivation, extend the observation period, establish sampling points across multiple timescales, and focus on the following areas: (1) Continuously monitor CO₂-C releases during the cultivation period to establish a comprehensive carbon balance; (2) Use ^13^C or ^14^C isotope tracing to distinguish the fate of exogenous carbon from that of soil-derived carbon; (3) Integrate data on microbial community composition, biomass, and functional traits to elucidate the regulatory mechanisms by which soil amendments modulate microbial communities and carbon and nitrogen cycling functions. The ultimate goal is to establish a systematic research framework spanning from microscopic mechanisms to the field scale.

## 5 Conclusion

Under short-term cultivation conditions, the treatment combining organic fertilizer and limestone powder (T3) exhibited the highest SOC content, significantly increasing soil pH as well as the content of amorphous Fe/Al oxides. Both T2 and T3 significantly increased the carbon content of microbial necromass. A two-factor analysis of variance revealed a significant interaction between organic fertilizer and limestone powder on POC, MAOC, MNC, and Fe/Al oxide forms, indicating that the two components exert an interactive regulatory effect on carbon fraction distribution and mineral phase transformation. Linear regression and correlation analyses showed that microbial necromass carbon and Fe/Al oxides (Fe_o_, Al_o_, Fe_p_) were significantly positively correlated with SOC and its fractions, suggesting close associations among microbial necromass carbon accumulation, Fe/Al oxide protection, and changes in SOC fractions. VPA indicated that microbial and mineral variables shared a high proportion of variance in explaining SOC fraction variation. Based on the observed changes in SOC and its fraction concentrations under short-term incubation, this study proposes the following conceptual hypothesis: microbial transformation and Fe/Al mineral retention may jointly participate in SOC accumulation in rubber plantations; although the two share statistical overlap, their causal pathways and relative contributions require further verification. These results provide short-term experimental references for regulating soil carbon pools in acidic rubber plantations through the combined application of organic fertilizer and limestone powder.

## Data Availability

The datasets presented in this study can be found in online repositories. The names of the repository/repositories and accession number(s) can be found in the article/supplementary material.
